# Macromolecular characterization of high β-glucan oat lines

**DOI:** 10.1016/j.heliyon.2024.e24552

**Published:** 2024-01-17

**Authors:** Abhinav Majumdar, Aina Belén Gil-González, Anna Barjuan Grau, Roya R.R. Sardari, Olof Larsson, Aishwarya Thyagarajan, Andreas Hansson, Oswaldo Hernández-Hernández, Olof Olsson, José Alfredo Zambrano

**Affiliations:** a Division of Pure and Applied Biochemistry, Lund University, Box 124, SE 22100, Lund, Sweden; b Department of Biotechnology, Lund University, Box 124, SE 22100, Lund, Sweden; c CropTailorAB, c/o Department of Pure and Applied Biochemistry, Lund University, Box 124, SE 22100, Lund, Sweden; d Institute of Food Science Research (CIAL), CSIC-UAM, c/Nicolás Cabrera, 9, 28049, Madrid, Spain

**Keywords:** *Avena sativa*, Dietary fibers, β-Glucan, Macromolecular quantifications, Amino acid analysis, Molecular weight determination

## Abstract

Oat (*Avena sativa*) is a cereal grain rich in fibers, proteins, vitamins and minerals. Oats have been linked to several health benefits, such as lowering blood cholesterol levels, counteracting cardiovascular disease and regulating blood sugar levels. This study aimed to characterize two new oat lines with high β-glucan content emanating from ethyl methyl sulphonate mutagenesis on the Lantmännen elite variety Belinda. Two of the mutated lines, and the mother variety Belinda, were profiled for β-glucan, arabinoxylan, total dietary fiber and starch composition. In addition, total lipid and protein content, amino acid composition and β-glucan molecular weights were analyzed. The high levels of β-glucan resulted in a significant increase in total dietary fiber, but no correlation could be established between higher or lower levels of the assayed macromolecules, i.e., between arabinoxylan-, starch-, lipid- or protein levels in the mutated lines compared to the reference. The results indicate separate biosynthetic pathways for β-glucans and other macromolecules and an independent regulation of the different polysaccharides studied. Therefore, ethyl methyl sulphonate mutagenesis can be used to increase levels of multiple macromolecules in the same line.

## Graphical abstract

**Figure:**
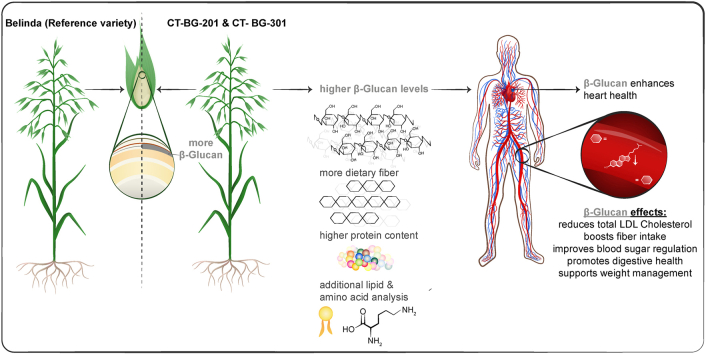

## 1 Introduction

Oats (*Avena sativa*) are cereals that are predominantly cultivated in Europe and North America, as the climatic conditions in these regions augur well for their growth [1]. Traditionally oats have mainly been used as feed, but human oat consumption is on the rise due to the fact that long-term oat consumption has proven to give several health benefits [2]. In addition, since oat is a crop with few diseases and it is an excellent rotation crop for wheat and oil seed plants. Therefore, an increased oat production would contribute to a more sustainable agriculture [3].

Dietary fibers are plant-based macromolecules that are resistant to digestion by human alimentary enzymes [4]. Typical dietary fibers are polysaccharides like resistant starch, cellulose, β-glucan, arabinoxylan (AX), inulin, mannan, etc. Non-polysaccharide dietary fibers like lignin also exist [5]. In comparison to their contemporaries in the cereal grain family, like wheat, barley and rice, oats are abundant in two crucial dietary fibers related to human health; β-glucans and AXs [6,7].

In addition, oats have a favorable lipid composition, proteins with a well-balanced amino acid composition and considerable amounts of vitamins, essential dietary minerals, and unique bioactive compounds like the polyphenol avenanthramide [2,8]. Thus, the nutritional composition of oats is well-rounded [9]. Because of this, a regular intake of oats will counteract negative health conditions like cardiovascular diseases, diabetes, hypertension, and obesity, among others [1].

The present registered health claims related to oats have been made in connection to its β-glucans [10]. According to the claims there is a cause-and-effect relationship between a daily consumption of β-glucan and a reduction of blood cholesterol concentrations, reduction of the glucose rise after a meal and an increase in the fecal bulk [[11], [12], [13], [14]].

β-Glucan is primarily present in the soluble fraction of the oat dietary fibers [15]. It is a hydrocolloid polysaccharide composed of d-glucose monosaccharides. The monomers are linked together with either β (1 → 4) or β (1 → 3) glycosidic linkage [16]. The primary component of the oat soluble fiber is arranged in such a way that the (1 → 4) linked β-d-glucose form continuous blocks that are separated by the (1 → 3) linkage. The molecular weight usually varies but normally occurs in a range from 20 thousand to 3 million Dalton [15,17]. In oats, most of the β-glucan is present in the endosperm cell walls, mainly in the thick sub-aleurone layer, although it is also present in the aleurone layer, albeit to a lesser extent [6,16].

Important physical properties of β-glucans like viscosity, solubility and water holding capacity vary depending on concentration, solubility, molecular weight, and tri-saccharide/tetra-saccharide ratio (DP3/DP4 ratio), which is around 2.4 to 2.6 in oats [16,18,19]. At lower concentrations, oat β-glucans tend to form more viscous solutions due to an interaction between different β-glucan molecules [20]. Because of their ability to form viscous and shear thinning solutions, β-glucans have a high-water holding capacity and therefore β-glucans are often used as thickening agents in different food formulations and in cosmetics [19].

β-Glucans have also proven prebiotic activities and positively affect our microbiota as these fibers act as substrates for health-promoting microbes in our intestinal microflora. For example, microbes can produce short-chain fatty acids that function as signal molecules and interact with specific receptors in the body. Among other things this results in a regulation of glucose and cholesterol levels in our bodies [21,22].

In addition to β-glucans, AXs are a part of the primary non-starch fraction of oat dietary fibers. In the past few years focus on the health benefits associated with regular consumption of AXs has increased [7,23]. In seeds, AXs are located in the cell wall of starchy endosperm (aleurone) in the bran tissue as well as in the husk [7]. The main structure consists of a β-1.4-d-xylopyranosyl backbone with ɑ-l-arabinofuranosyl residues substituted at positions 2 and/or 3 [24].

Compared to other frequently consumed cereal grains like wheat, rice and maize, the quality of oat proteins is superior due to a better-balanced amino acid profile [25]. This is true both when oat is consumed as whole seeds or as dehulled kernels [26]. Protein composition in plants is categorized into different classes based on their biochemical solubility, namely albumins (water-soluble), globulins (salt-water-soluble), prolamins (alcohol-soluble), and glutelin (acid or base soluble) [27]. Oat is unusual among cereals since almost 80% of the total proteins fall under the category of globulins [9].

This is positive from a health perspective since the amino acid distribution in the four protein fractions is markedly different and essential amino acids like lysine, histidine, valine, and phenylalanine are predominantly found in the globulin fraction [28,29].

Previously, random chemical ethyl methyl sulphonate (EMS) mutagenization was performed on the Lantmännen elite variety Belinda [30], to increase the genetic variability in the breeding material and pave the way for the unearthing of novel characteristics, ultimately leading to the development of new, superior oat varieties. EMS is an ethylating agent which, by inducing ethylation of guanidine, mainly converts guanine-cytosine (GC) base pairs in the DNA molecule to adenine-thymine (AT) base pairs [31,32]. From an industrial standpoint, this technique presents a great advantage in the development of new traits since they will not fall under the umbrella of Genetically Modified Organism (GMO). Thus, EMS-induced traits can be readily commercialized without the excessive safety testing required for GMOs [7,32]. Previously the oat mutagenized population has successfully been used to generate oat lines with high β-glucans-, proteins-, avenanthramides- and AXs levels as well as with lower lignin levels [7,16,[33], [34], [35]].

As mentioned, β-glucan is a vital dietary fiber that provides holistic health benefits and aids in the counteraction of common health disorders. A further biochemical characterization of additional essential polysaccharides in the previously identified high β-glucan lines [16] would help in accessing to what extent these molecules can provide extra health benefits. In addition, we wanted to investigate if the mutagenesis technique used gave rise to any unexpected structural modifications of other polysaccharides present in oats.

The objective in this study was to characterize both the β-glucan in chosen high β-glucan lines and additional macromolecules, more specifically AXs, lipids and proteins to find out if elevated levels of one of these macromolecules somehow affected the levels of the others. If no negative interactions occur, this would encourage a more systematic future breeding for varieties where several important macromolecules have simultaneously been increased, i.e., to produce even more healthy oat varieties than the present ones.

## 2 Materials and methods

### 2.1 Oat samples

The experimental oat lines, CROPTAILOR-BG-201 (CT-BG-201) and CROPTAILOR-BG-301 (CT-BG-301), used in this study were developed and provided by CropTailor AB (www.croptailor.com). CropTailor maintains the EMS mutagenized oat population based on the Lantmännen’s oat variety Belinda which was developed by Chawade et al. [30]. Individual lines from this population are denoted as CropTailor (CT)-lines.

CT-BG-201 and CT-BG-301 are the results of the breeding program in CropTailor’s β-glucan project with the aim of stacking mutations which has a positive effect on β-glucan content. Two CT-lines identified as high in β-glucan were crossed together, and a half-seed analysis technique was used to select the best individual seeds from the F2 generation and in succeeding F-generations [36]. These early generations were grown in greenhouse and climate chambers. As variation in growth conditions is a contributing factor to the absolute β-glucan content of seeds from a given growth cycle the parent variety Belinda was grown alongside each F-generation as a control. Results from control measurements show that the β-glucan lines used here had an average of 58.93% higher β-glucan content than the Belinda control over the first 5 F-generations.

The final oat samples provided for analysis and used in the present study were later F-generation accessions of field-grown material grown at Lönnstorp Research Station in 2020 where the Belinda sample included in the study was also grown at the same field trial.

### 2.2 Complex carbohydrate analysis

Several different complex carbohydrates were analyzed in this study including total dietary fiber (TDF), starch, AX and β-glucan. The Megazyme^Tm^ Total Dietary Fiber kit (K-TDFR) was used to determine TDF content of oat samples. Amylase, protease, and amyloglucosidase enzymes are included in the kit. The dietary fiber content of the soluble and insoluble extracts was determined using the Megazyme^Tm^ online calculator, which eliminates proteins and ash. TDF is the sum of the soluble and insoluble fiber fractions. The total starch content was analyzed according to the protocol adapted from the Megazyme^Tm^ Total Starch Assay kit (K-TSTA). The method employs two enzymes for the breaking down of complex branched starch chains into linear chain maltodextrins followed by hydrolyzation of the resultant maltodextrins into simpler glucose units. Thermostable α-amylase was used for the former and amyloglucosidase was used for the latter. The resultant glucose produces a pink color upon reaction with GOPOD reagent, which was quantified by spectrophotometrically measuring absorbance at 510 nm. A glucose standard was used for comparison for the estimation of total starch. The assay was scaled down to one-fourth of the reagent used, with minimal to no compromise on assay efficiency and accuracy.

The non-starch carbohydrate, β -glucan was estimated in a similar manner using the β -glucan assay kit from Megazyme^Tm^ (K-BGLU) with minor modifications. The complex β -glucan was broken down into simple d-glucose through a series of enzymatic digestion using the enzymes lichenase and β-glucosidase and the absorbance of the resultant glucose was measured as described above.

AX, a hemicellulose made up of arabinose, xylose and galactose was measured as described by Sardari et al. with some minor modifications [37]. The polysaccharide was subjected to a brief acid hydrolysis using 72% sulfuric acid and incubated in a shaker incubator at 30 °C at 200 rpm to which water was added after 1 h. The samples were then heated to 100 °C for 3 h (including warm up and cool down). This was followed by neutralization using 0.1 M Ba (OH)_2_·H_2_O. The supernatant was collected and filtered using 0.2 mm syringe filter in HPLC vials and monosaccharide analysis was done using a High-Performance Anion-Exchange Chromatography (HPAEC) system (Thermofisher Scientific, Waltham, USA) using a Dionex CarboPac PA-20 analytical and PA-20 guard columns. The monosugar separation was carried out using 0.75 mM NaOH at a flow rate of 0.5 mL/min. The column regeneration was performed using a higher concentration of NaOH (200 mM) for 4 min at the end of each cycle with the same flow rate. An ED40 electrochemical detector which measures the current that flowthrough cell electrodes produce when potential (voltage) is applied across them, was used for the detection of peaks.

### 2.3 Molecular weight determination of β-glucan

β-Glucans molecular weights were determined essentially as previously described [38]. In the first step, β-glucans were extracted from the oat samples by boiling in 50% ethanol in a closed container for 15 min followed by transfer to 20 mL of hot deionized water. To this, CaCl_2_ (0.28 mg/mL of water) was added along with 50 μL of thermostable α-amylase. The solution was incubated in a boiling water bath for 90 min with occasional stirring after which the solution was centrifuged at 1500*g relative centrifugal force (RCF) for 15 min. The pellets collected at this stage were solubilized in pH 6 sodium phosphate buffer. This solution was subsequently incubated overnight with 1 U/mL xylanase (Megazyme E-XYRU6) at 30 °C to get rid of any water-soluble AX. The remaining xylanase was then inactivated by heat shock treatment at 100 °C for 5 min, after which the solution was filtered through 0.45 μm syringe filters and transferred into chromatography vials.

Size Exclusion Chromatography (SEC) was performed on an Agilent 1260 II liquid chromatographer (Agilent Technologies, Santa Clara, CA, USA) consisting of an isocratic pump, an oven, and an autosampler with a 100 μL loop. The isocratic pump module delivered 1 mL/min of an aqueous solution containing 200 mM NaNO_3_ and 10 mM NaH_2_PO_4_, which were sterile filtered through a 0.22 μm filter. The solution was passed through two PL aquagel-OH MIXED-H columns (8 μm, 300 × 7.5 mm) connected in series.

The detection system (Agilent Multi-Detector GPC/SEC) included a dual light scattering setup (15° and 90°) equipped with a λo = 658 nm laser, a refractive index detector and a viscometer. Calibration was performed using polyethylene glycol/oxide standards (InfinityLab EasiVial PEG/PO, Agilent Technologies) with a range of Mw 106 to 1,500,000 g/mol. The refractive index increment (dn/dc) used was 0.146, which is commonly used in aqueous solutions of polysaccharides. The calibration was tested using Mw 265,000 and 391,000 g/mol β-glucan standards from oat (Megazyme-Neogen, Lansing, MI, USA).

Data acquisition and analysis were performed using GPC/SEC Software from Agilent Technologies. Molecular weight averages are expressed as the number average (Mn), the weight average (Mw) and z-average molecular weight (Mz) defined by the following formulas [39]:

$${Mn=(\Sigma N}_{i}M_{i}{)\;/\;(\Sigma N}_{i})$$

Mw=(ENiMi2)/(ENiMi)

Mz(ENiMi3)/(ENiMi)

where Ni is the number of chains and Mi is the molecular weight of each unique chain.

The polydispersity index (PD), which indicates the broadness of the molecular weight distribution, is calculated as:

PD=Mw/Mn

### 2.4 Protein measurement

Total protein content was quantified by measuring total nitrogen present in the samples by means of the Dumas method [40]. Approximately, 25 mg of milled oat flour from each line was weighted in a foil disc and analyzed. Aspartic acid was used as the standard for this method and the final protein content was calculated using a Jones conversion factor of 6.25 [34,41,42].

### 2.5 Amino acid composition

The analysis of the amino acids profile of Belinda and of the CropTailor high β-glucan lines (CT-BG-201, CT-BG-301) was performed using a recently described procedure [43]. Briefly, flour samples were first defatted in a chloroform-methanol mixture and then liquid-phase acid hydrolysis was performed on dried defatted flour samples (1 mg) suspended in 1 mL 6 M HCl supplemented with 0.1% (w/v) phenol. The hydrolysis was performed by incubating at 110 °C in a Techne Dri-Block heating block (Cole-Parmer, Vernon Hills, USA) for 24 h. The acid was then removed from the hydrolyzed sample using the Techne sample concentrator (Cole-Parmer). Subsequently, the amino acids were reconstituted in 1 mL (same volume as the HCl solution used for hydrolysis) of l-norleucine solution (0.5 mM) in ultrapure water and filtered through a 0.2 μm polypropylene filter. After this, the amino acids were injected into an HPAEC system (ThermoFisher Scientific, Waltham, USA) with a Dionex AminoPac PA10 analytical column coupled to a Dionex AminoPac PA10 guard column (ThermoFisher Scientific) [44]. All the samples contained 0.5 mM l-norleucine as an internal standard. In addition, a standard amino acids mix solution (TraceCERT grade certified reference material; Sigma-Aldrich, Sweden) was included. This mix contained 2.5 mM each of l-arginine, l-lysine, l-alanine, l-threonine, glycine, l-valine, l-serine, l-proline, l-isoleucine, l-leucine, l-methionine, l-histidine, l-phenylalanine, l-glutamic acid, l-aspartic acid and l-tyrosine and 1.25 mM of l-cysteine. The HPAEC system included four different eluents: pump A (ultrapure water), pump B (1 M sodium acetate), pump C (250 mM NaOH) and pump D (100 mM acetic acid). Separation occurred using gradient conditions at a flow rate of 0.25 mL/min and during a running time of 74 min. The analytes were detected with an ED40 electrochemical detector (AAA-certified disposable gold electrode) (ThermoFisher Scientific).

For the tryptophan analysis, alkaline hydrolysis of the dried defatted flour samples was done using 4.2 M NaOH as hydrolysis solution. One milligram of the sample was weighed into the glass tube and suspended in 400 μL of NaOH. After purging with nitrogen, the glass tube containing the sample was screwed tightly and incubated at 110 °C for 24 h. Hydrolyzed samples were processed for analysis after proper dilution. l-tryptophan standards with different concentrations were prepared separately.

The standards of cysteic acid, methionine sulfoxide, methionine sulfone (Sigma-Aldrich, Sweden) were used separately and the analysis of these oxidation products in the samples was carried out using the same method described above simultaneously with the amino acids. The mass fraction of released amino acids (AA) were used for the calculation of the original mass fraction of amino acids in the sample (AA0) as described by Sardari et al. [43].

### 2.6 Lipid content

Total oat kernels lipids from Belinda and the mutated lines (CT-BG-201 and CT-BG-301) were quantified by first grinding the kernels to a fine flour and then extracting the lipids from the flour with a mixture of heptane and isopropanol at a ratio of 3:2. Efficient percolation of the solvent into the flour particles was facilitated using a mixer (Precellys® Evolution) at 5000 rpm 5 × 90 s. After the extraction the solution was centrifuged at 9000 rpm in an MPW-352R benchtop refrigerated centrifuge. The supernatant containing the lipids was rescued and after 48h of evaporation of the solvent at room temperature, the total weight of the remaining lipids was measured and calculated as a percentage of total flour weight [45].

### 2.7 Statistical analysis

A two Sample *t*-test and an ANOVA test were performed to determine a significant difference between samples for the levels of β-glucan, protein, and the other macromolecules between the mutated lines (CT-BG-201 and CT-BG-301) and the reference variety (Belinda). The Spearman rank correlation coefficient was employed as a robust statistical measure to assess the strength and direction of monotonic relationships between the variables. All the results obtained after using technical replicates.

## 3 Results

### 3.1 Carbohydrate profile

TDF, β-glucan, AX, and starch were quantified in the mutagenized high-glucan lines, as shown in Table 1, along with the reference line Belinda. This confirmed that the average concentration of β-glucan in both the mutagenized lines was higher than in the reference variety Belinda. The mutagenized lines CT-BG-201 and CT-BG-301 contained 6.46% (DW) and 5.81% (DW) β-glucan on a dry basis, respectively, in comparison to Belinda, which had 4.43% (DW). These results show a statistically significant (*p*-value < 0.05) increase of 46% and 31% in both lines, confirming that both mutated lines have high levels of β-glucan. Similar analyses of AX revealed that in contrast to Belinda’s 4.18% (DW), CT-BG-201 and CT-BG-301 had 3.68% (DW) and 3.04% (DW), respectively, of AX. A simultaneous quantification of β-glucan and AX adds to a deeper knowledge of the nutritional profile of a certain line. In the two high β-glucan lines analyzed here, no relationship between AX- and β-glucan levels could be established.

**Table 1:** Complex carbohydrate and total protein contents of Belinda, CT-BG-201, and CT-BG-301. Results are presented as means ± SD (p < 0.05).

	Oat Lines		
**Analyzed macromolecules**	**Belinda**	**CT-BG-201**	**CT-BG-301**
**Soluble dietary fiber (% w/w)**	3.40 ± 0.28	5.79 ± 1.30	4.10 ± 1.45
**Total starch (% w/w)**	44.05 ± 3.81	35.41 ± 3.11	39.53 ± 1.18
**Total lipid (% w/w)**	6.95 ± 0.04	7.05 ± 0.08	7.25 ± 0.12
**Total protein (% w/w)**	11.25 ± 0.27	16.59 ± 0.07	16.56 ± 0.06
**Total β-glucan (% dw)**	4.43 ± 0.17	6.46 ± 0.05	5.82 ± 0.27
**Total AX (% w/w)**	4.18 ± 0.15	3.68 ± 0.34	3.04 ± 0.14
**TDF (% w/w)**	10.19 ± 0.82	15.50 ± 2.01	13.28 ± 0.33
**Insoluble dietary fiber (% w/w)**	6.80 ± 1.11	9.71 ± 0.71	9.18 ± 1.12

On the other hand, both the CT-BG-201 and CT-BG-301 lines had a higher total TDF content compared to the reference line Belinda. Here we define TDF as a summary of the insoluble and soluble dietary fiber fractions. In the two high β-glucan lines the average TDF content was 15.50% (W/W) and 13.28% (W/W) respectively, as compared to 10.19% (W/W) in Belinda. These higher TDF levels in the high β-glucan lines are most likely due to their higher β-glucan amount.

The total starch was also analyzed, and in this case, both the mutagenized lines had slightly lower content compared to Belinda. The total starch was 35.42% for CT-BG-201 and 39.53% for CT-BG-301, while Belinda contained 44.05%.

### 3.2 Molecular weight of β-glucan

Our results demonstrate that one of the two mutated CropTailor lines (CT-BG-201) exhibited a β-glucan molecular weight (Mw) similar to that of Belinda (Table 2) but with a significantly higher polydispersity index (2.855 vs. 1.747). Conversely, CT-BG-301 showed a similar polydispersity index but a much higher β-glucan Mw value compared to Belinda or CT-BG-201. Thus, in our experiments, the differences in Mw are not proportional to the β-glucan content (Table 1, Table 3) [38,46].

**Table 2:** Molecular weight averages (Mn, Mw and Mz) and polydispersity indexes (PD) for Belinda, CT-BG-201, and CT-BG-301 (p < 0.05).

Oat Lines	Mn (g/mol)	Mw (g/mol)	Mz (g/mol)	PD
Belinda	176,038	307,466	643,825	1.747
CT-BG-201	146,105	417,094	1,557,274	2.855
CT-BG-301	644,732	1,120,259	1,851,599	1.738

**Table 3:** The amino acid profile of defatted flours (Belinda, CT-BG-201, and CT-BG-301) determined by direct analysis of amino acids and subsequently calculated by nonlinear least-squares regression analysis. Columns 5 and 6 refer to FAO’s recommended amino acid scoring pattern for infants (birth to 6 months) and children (6 months–3 years) [47]. Results are presented as means ± SD (p < 0.05).

	Belinda	CT-BG-201	CT-BG-301	FAO, infant	FAO, Child
Amino acid	(%)	(%)	(%)	mg/10 g protein requirement	
**L-Ala**	6.6 ± 0.77	7.27 ± 1.9	7.3 ± 1.12		
**L-Asp + L-Asn**	8.9 ± 0.66	8.25 ± 1.16	8.3 ± 0.51		
**L-Cys + Cysteic acid**	2,9 ± 1.73	2.64 ± 1.67	2.5 ± 0.32		
**L-Glu + L-Gln**	8.4 ± 0.36	7.67 ± 0.89	8.4 ± 0.58		
**Gly**	6.7 ± 1.69	7.11 ± 2.63	7.5 ± 1.58		
**L-His**	3.8 ± 0.74	3.48 ± 0.81	3.6 ± 0.29	2.1	2.0
**L-Ile**	6.0 ± 0.84	6.72 ± 1.2	6.4 ± 1.42	5.5	3.2
**L-Leu**	11.4 ± 3.27	10.87 ± 0.48	10.9 ± 1.47	9.6	6.6
**L-Lys**	6.5 ± 1.23	6.05 ± 1.03	5.4 ± 0.29	6.9	5.7
**L-Met**	1.3 ± 0.4	2.03 ± 0.57	1.7 ± 0.91		
**Methionine sulfoxide**	0.6 ± 0.35	0.26 ± 0.2	0.3 ± 0.29		
**L-Phe**	6.5 ± 0.45	7.16 ± 1.53	7.6 ± 0.94		
**L-Pro**	3.8 ± 1.02	4.02 ± 1.29	4.02 ± 1.29		
**L-Ser**	7.8 ± 2.09	7.12 ± 2.4	6.9 ± 0.87		
**L-Thr**	5.5 ± 1.37	5.36 ± 1.79	5.1 ± 0.6	4.4	3.1
**L-Trp**	0.4 ± 0.21	0.47 ± 0.07	0.6	1.7	0.85
**L-Tyr**	6.9 ± 0.9	6.06 ± 1.16	6.2 ± 0.44		
**L-Val**	7.0 ± 1.32	8.53 ± 1.78	8.3 ± 0.73	5.5	4.3

In this study, three non-selective detectors (RI, viscometer, and light scattering) were employed in the molecular weight measurements. As a result, a comprehensive β-glucan fractionation was conducted, which included enzymatic hydrolysis of starch, AXs, and proteins to mitigate interference from these polymers in the analysis. The combination of these three detection methods allowed for the calculation of different molecular weight averages, specifically Mn, Mw, and Mz. These values enable us to understand the β-glucan molecular weight distributions per sample. Table 2 shows that the polydispersity index was similar in Belinda and the CT-BG-301 line. In comparison, CT-BG-201 molecular weights were highly dispersed in comparison to the other two oat lines, meaning that there is a broad range of molecular weights in this sample.

Despite having a similar polydispersity value, Belinda and CT-BG-301 oat lines have different molecular weight averages. In general, Belinda has much lower molecular weight values than CT-BG-301. In fact, Belinda’s Mz value, which represents the higher average molecular weights, is similar to CT-BG-301's Mn value, which represents the total molar weight divided by the total number of β-glucan molecules. CT-BG-201 has a lower Mn value; however, the Mw and Mz values are higher than Belinda, particularly the Mz value, indicating that the influence of higher molecular weights is more pronounced in CT-BG-201 than in Belinda. In general, we can consider that CT-BG-301 has the highest molecular weight values, followed by CT-BG-201 and Belinda.

### 3.3 Protein quantification

The protein content in flour was analyzed in the dehulled and milled oat kernels both in the mutagenized high β-glucan lines and in the Belinda control variety. This showed that the CropTailor lines had higher protein levels compared to Belinda. The estimated protein content for Belinda was 11.25% while for the mutagenized lines, it was 16.59% and 16.56% respectively, i.e., a 47% increase (Table 1). Thus, both the high β-glucan lines turned out to have a higher protein content compared to Belinda. However, we have no evidence that these traits are genetically related so at this point it is only an observation, not a proof of causality.

### 3.4 Amino acid analysis

The amino acid composition in proteins isolated from Belinda and the mutagenized lines CT-BG-201 and CT-BG-301 are shown in Table 3. The amino acid l-arginine could not be determined due to co-elution with unknown compounds. The total amino acids of the sample Belinda, CT-BG-201 and CT-BG-301 were 11.5, 13.8, and 12.6% of flour, respectively. These values are in general lower than the total protein values given in section 3.3 and Table 1. Reasons for this could be the differences in the analytical methods, loss of proteins during the defatting step or overestimation of the protein content due to the protein conversion factor of 6.25 used.

The data in Table 3 show that proteins from both the mutagenized lines and the reference line Belinda contained all nine essential amino acids i.e., the amino acids that the human body cannot synthesize and thus must be obtained through diet (Table 3). Furthermore, the proportions between the different amino acids were similar in the lines with higher protein levels when compared to Belinda. A recent study has determined that the average daily protein requirement for adults is estimated to be 0.91 g of protein per kilogram of body weight [47]. Thus, regular oat consumption will provide the essential amino acids necessary to maintain muscle mass and for overall health and wellness.

### 3.5 Lipid analysis

The total content of lipids present in the mutagenized oat lines together with the reference oat variety Belinda was studied by quantifying the amount of lipids in dehulled and milled oat kernels. It was found that the average lipid concentration for both the mutagenized lines was slightly higher than for Belinda. The mutagenized line CT-BG-201 had an average lipid content of 7.05% (w/w), as compared to Belinda, which had an average lipid content of 6.95% (w/w) (Table 1). The other mutagenized line (CT-BG-301) had even higher levels, i.e., a significant increase of 4% of the total content of lipids compared to Belinda, with a 7.24% (w/w) of average lipid concentration.

## 4 Discussion

Oats have been associated with beneficial health properties for a long time. In recent years abundant scientific evidence has accumulated that confirms this notion. It has also become clear that a healthy diet to counteract welfare diseases like type 2 diabetes and cardiovascular disease is becoming increasingly important with a global population of more and more elderly people. Food is a key to health, and it is therefore of utmost importance to develop novel foods with enhanced nutritional and health-promoting characteristics. In this development, oats will have an important role. However, in this work, it will be crucial to determine that a specific genetic event that results in the improvement of one such component will not interfere with or counteract another.

In this study, two oat lines denoted CT-BG-201 and CT-BG-301, developed by crossing two lines from the Belinda [30] mutant population, were used as a model to investigate possible macromolecular interactions. These lines were initially chosen because of their high β-glucan content.

A carbohydrate profile was prepared for the reference variety Belinda and 2 chosen oat high β-glucan lines (CT-BG-201 and CT-BG-301) by characterizing their amount of β-glucan, AX, TDF and total starch. The analysis showed, as expected, significantly elevated levels of β-glucan in both the CT-lines, corroborating the basis for the preliminary screening of these lines. The higher β-glucan levels also resulted in a significant increase in the amounts of TDF, especially in the soluble fraction. However, no correlations or any other systematic variations in levels of AX, total starch, lipids, and proteins were observed in the CT-lines in relation to the reference Belinda variety, strongly suggesting that their levels are regulated independently. Thus, the biosynthetic pathways for β-glucans and the other macromolecular are not overlapping and are not using the same precursors or other essential elements that will limit the biosynthesis of the others.

As β-glucans are currently the only molecules with a health claim, we also analyzed the molecular weights of β-glucans in the two mutated high β-glucan lines. Molecular weight is an important factor as studies have shown that higher molecular weights result in increased viscosity, and viscosity plays a key role in mediating the hyperglycemic and hypercholesterolemic effects of β-glucans by binding to cholesterol in the gut and promoting its excretion [48]. Our experiments indicate that higher molecular weight of β-glucans is not proportional to higher concentrations of β-glucans, which contrasts with previous reports [38]. However, this positive correlation has not been further elucidated and remains poorly understood [46].

A previous study reported β-glucan Mw values for Belinda around 2000 kDa [16]. Discrepancies in these values are attributed to diverse factors, including environmental effects on seed development and variations in analytical procedures. Environmental conditions can contribute significantly, accounting for up to 71% of β-glucan Mw differences [38]. The chosen analytical method also influences molecular weight values. For instance, the widely used SEC coupled with fluorescence detection and calcofluor derivatization can lead to an overestimation of the molecular weight average, especially beyond the 10,000 to 500,000 g/mol range. Outside this range, the calcofluor method provides apparent molar mass values due to β-glucan aggregation, which can be disrupted by shear forces from SEC columns and viscometer capillaries [49]. Discrepancies in analytical methods have been extensively examined in an inter-laboratory study using commercial β-glucan extracts [50].

Sikora et al. [16] utilized the calcofluor method, treating oat flour with NaOH and ddH2O, and analyzing the solution via SEC. Discrepancies in Mw values reported by Sikora et al. [16] and this study are likely due to differences in analytical methods.

The CT-BG-301 line exhibits a higher average molecular weight of β-glucan compared to the reference variety Belinda, while maintaining a similar polydispersity index. Higher polydispersity and lower molecular weights in β-glucans generally lead to quicker gel formation, and breaking these gels requires a higher torsion force. This rheological parameter could potentially influence physiological outcomes when β-glucans are used as functional ingredients.

Notably, studies linking β-glucan molecular weights to health properties often rely on weight average molecular weight (Mw) calculations. However, in cereal β-glucans, molecular weights are polydisperse, resulting in significant differences in lower or higher molecular weight averages even with similar Mw values. In this study, although Belinda and CT-BG-201 had a mere 110,000 g/mol difference in Mw, their Mz values differed by approximately 900,000 g/mol, indicating a pronounced presence of higher molecular weights in CT-BG-201. This suggests that molecular weight averages should be considered in food formulations and various in vivo studies due to their significant impact.

Our lipid quantifications showed that also the total lipid content was higher in the CT-BG-201 and CT-BG-301 lines compared to Belinda. High lipid content gives a higher energy value for the grain and is thus considered to be a positive character for feed. However, we have no biochemical evidence that there in general is a positive correlation between elevated β-glucan and lipid levels. Furthermore, low lipid content is also attractive in other applications, especially for milled oats. We have preliminary data showing that both high and low lipid lines can be found in the mutagenized population.

Total protein was also quantified, and the protein values were found to be higher in all three investigated oats in comparison to other cereal grains, as expected from previous investigations [9]. In addition, compared to other cereals, oat proteins have a higher proportion of water-soluble globular proteins, which contain all the nine essential amino acids, i.e., amino acids that the human body cannot synthesize and must be obtained through the diet. Thus, oats have both high protein levels and good quality proteins and will provide the body with the amino acids necessary to maintain muscle mass [25]. Our protein measurements showed that the CT-BG-201 and CT-BG-301 lines had slightly higher protein levels than Belinda. In addition, the amino acid distribution was analyzed, and it was found that the amino acids were uniformly distributed in Belinda and the two CT-lines although the mutated lines had a slightly higher total protein content. Thus, the elevated protein levels could not be explained by an unproportionate rise of specific single amino acids.

## 5 Conclusion

This study has successfully characterized 2 oat lines (CT-BG-201 and CT-BG-301) and one established oat variety (Belinda). This confirmed that the genetic modifications induced in the CT-lines resulted in significantly elevated levels of β-glucan. A deeper characterization revealed that the β-glucan molecular weight averages are in general higher in the mutagenized lines than that in Belinda, with a considerably higher molecular weight polydispersity in the line CT-BG-201 than Belinda and CT-BG-301. By further quantifying other macromolecules like AX, starch, fibers, lipids, and proteins, in Belinda and the two CT-lines, we show that their levels are regulated independently of each other. Thus, through EMS mutagenesis it is possible to individually increase the levels of multiple macromolecules at the same line, which opens up possibilities to develop more healthy oat varieties than those available at present. This finding, combined with the point that EMS-induced traits can be readily commercialized without the excessive safety testing required for GMOs [7,32,51], show that this technique is highly effective in creating increased genetic variation in the breeding material. As of 2022 CropTailor AB has developed one new variety from the mutagenized oat population. The variety, named Armstrong has passed DUS and VCU testing and has an increased value due to its high This study has shown that, through EMS mutagenesis-glucan, high protein and high oil content. It has now been added to the Swedish variety list, and CropTailor has been granted EU Plant breeder’s rights [52]. This demonstrates the viability of using lines from the mutant population as pre-breeding material for new variety development.

This study has shown that, through EMS mutagenesis, new oat lines presenting higher amounts of different macromolecules can be obtained. Oats presenting more β -glucans, AXs and other fibres, s well as other nutrients, will have a better positive impact on human health. Furthermore, as has previously been shown, the Mw of β -glucan Mw is also an important parameter that and needs to be considered in food formulation.

## Data availability statement

The authors confirm that the data supporting the findings of this study are available within the article and its supplementary materials.

## CRediT authorship contribution statement

**Abhinav Majumdar:** Writing – review & editing, Writing – original draft. **Aina Belén Gil-González:** Writing – review & editing, Formal analysis. **Anna Barjuan Grau:** Investigation. **Roya R.R. Sardari:** Writing – review & editing, Methodology. **Olof Larsson:** Methodology, Formal analysis. **Aishwarya Thyagarajan:** Methodology. **Andreas Hansson:** Writing – review & editing, Methodology. **Oswaldo Hernández-Hernández:** Writing – review & editing, Methodology. **Olof Olsson:** Writing – review & editing, Supervision. **José Alfredo Zambrano:** Writing – review & editing, Writing – original draft, Visualization, Supervision.

## Declaration of competing interest

The authors declare that they have no known competing financial interests or personal relationships that could have appeared to influence the work reported in this paper.
