# Supplementary material for: Macromolecular characterization of high β-glucan oat lines
Source: Heliyon. 2024 Jan 17;10(2):e24552. doi: 10.1016/j.heliyon.2024.e24552 (PMC10834800; doi:10.1016/j.heliyon.2024.e24552)
Supplement: Multimedia component 1 [file mmc1.docx]

Supplementary data


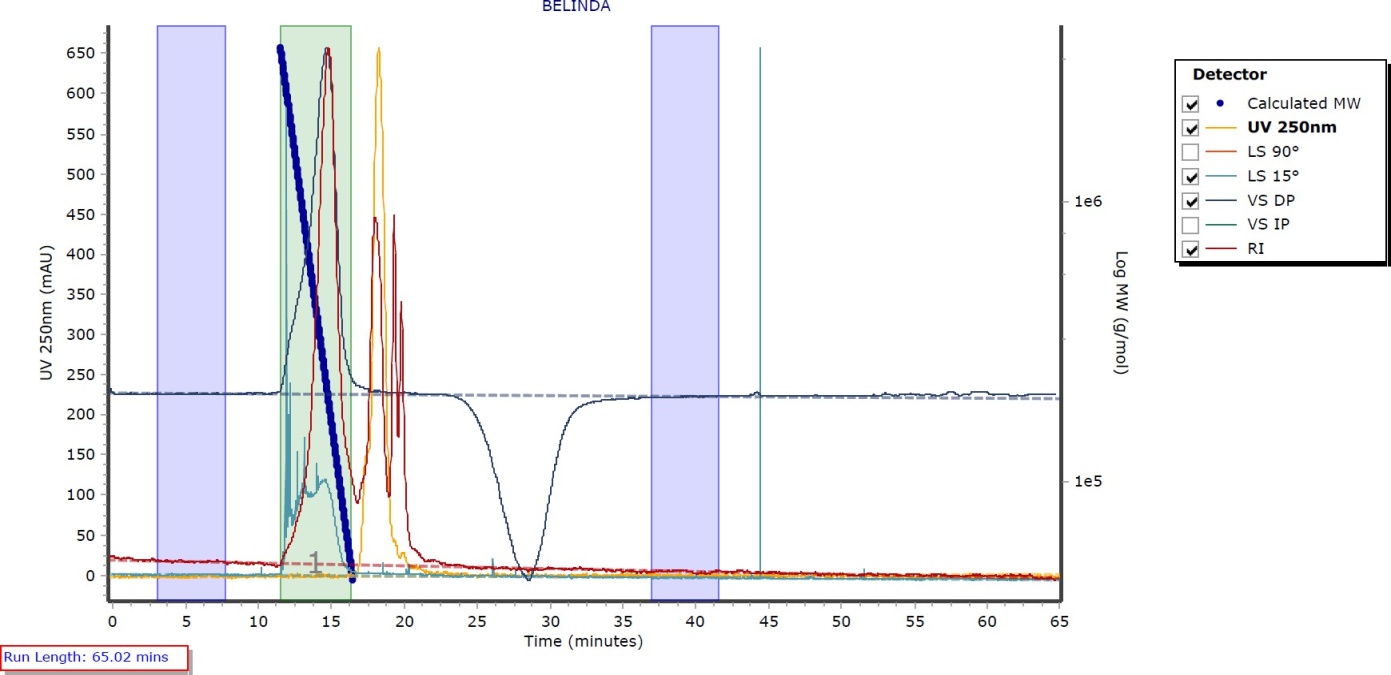
BELINDA


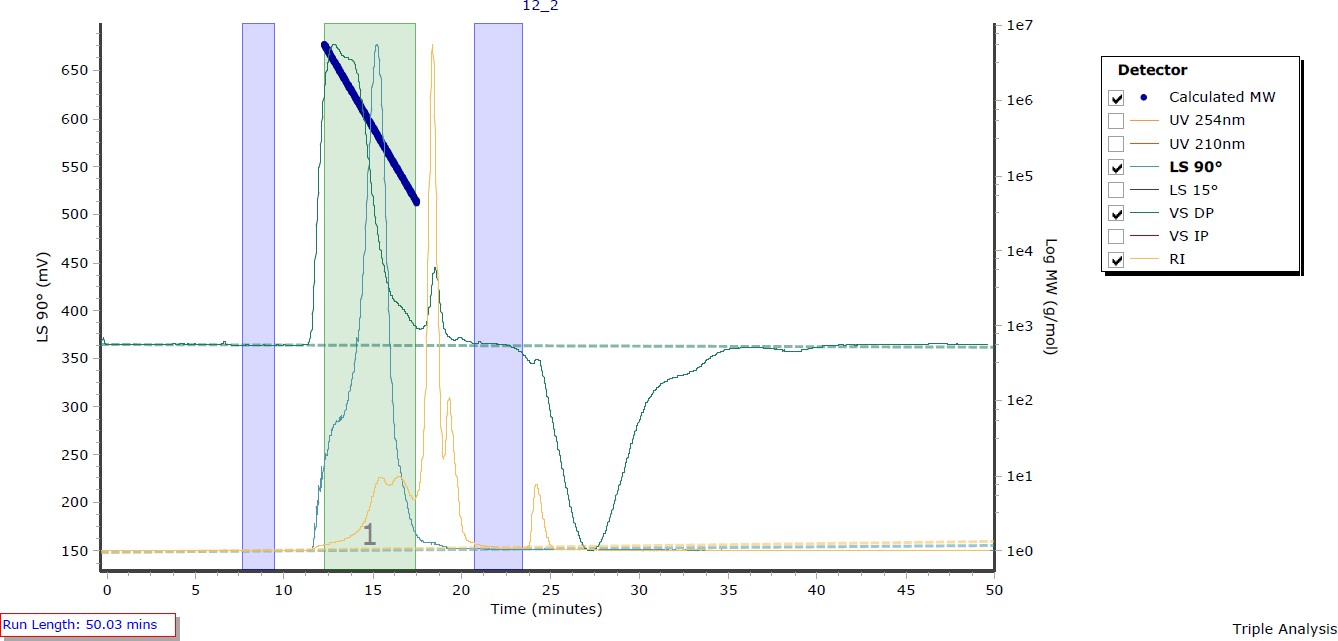
CROPTAILOR-BG-201


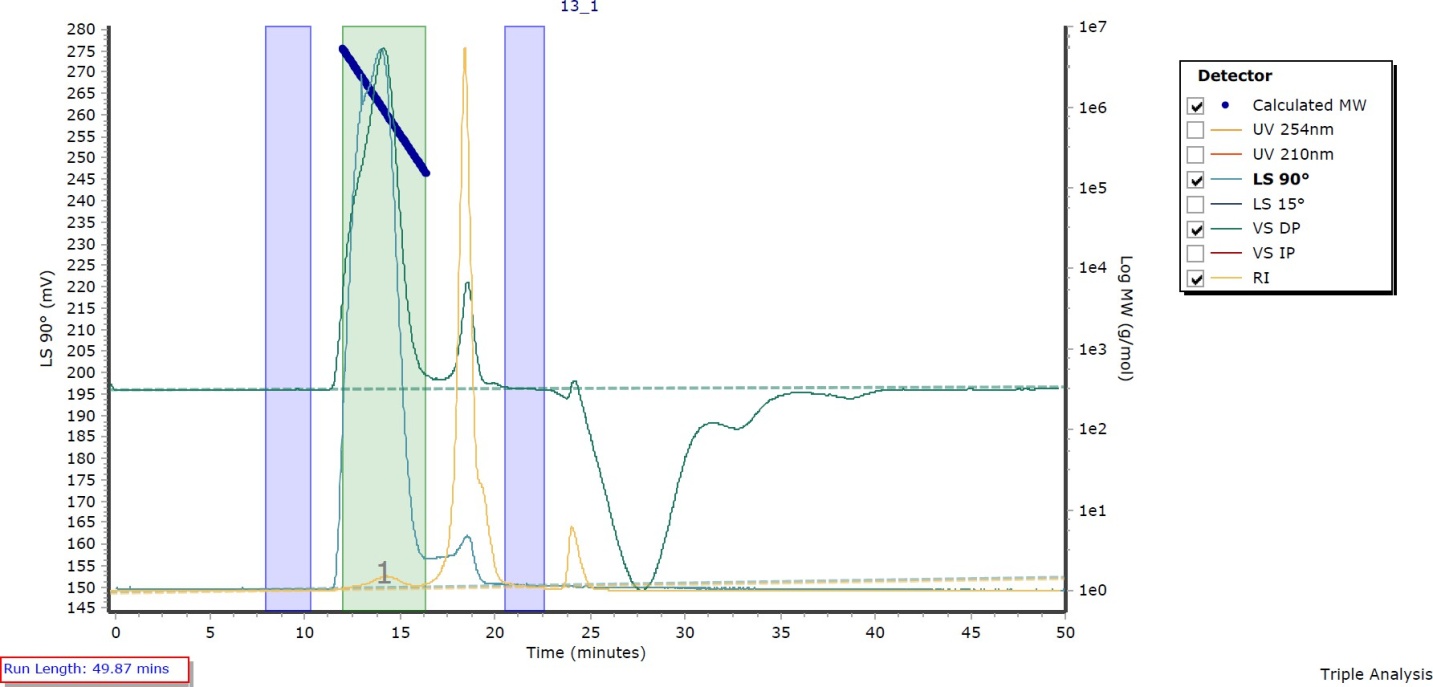
CROPTAILOR-BG-301


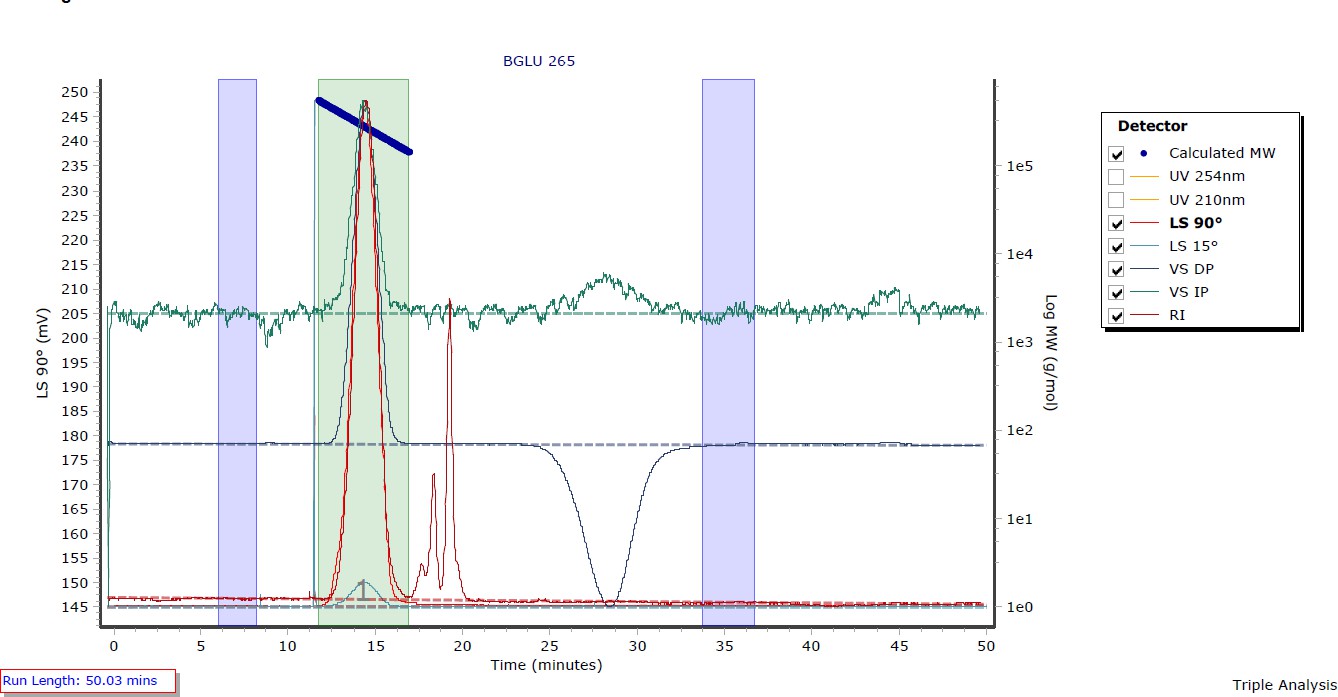


OATβ-GLUCANSTANDARD[Mw265,000]

*MwControl Sample*

*β-glucanMwstandards(Megazyme,P-MWBGS)*

BARLEYβ-GLUCANSTANDARD[Mw650,000]


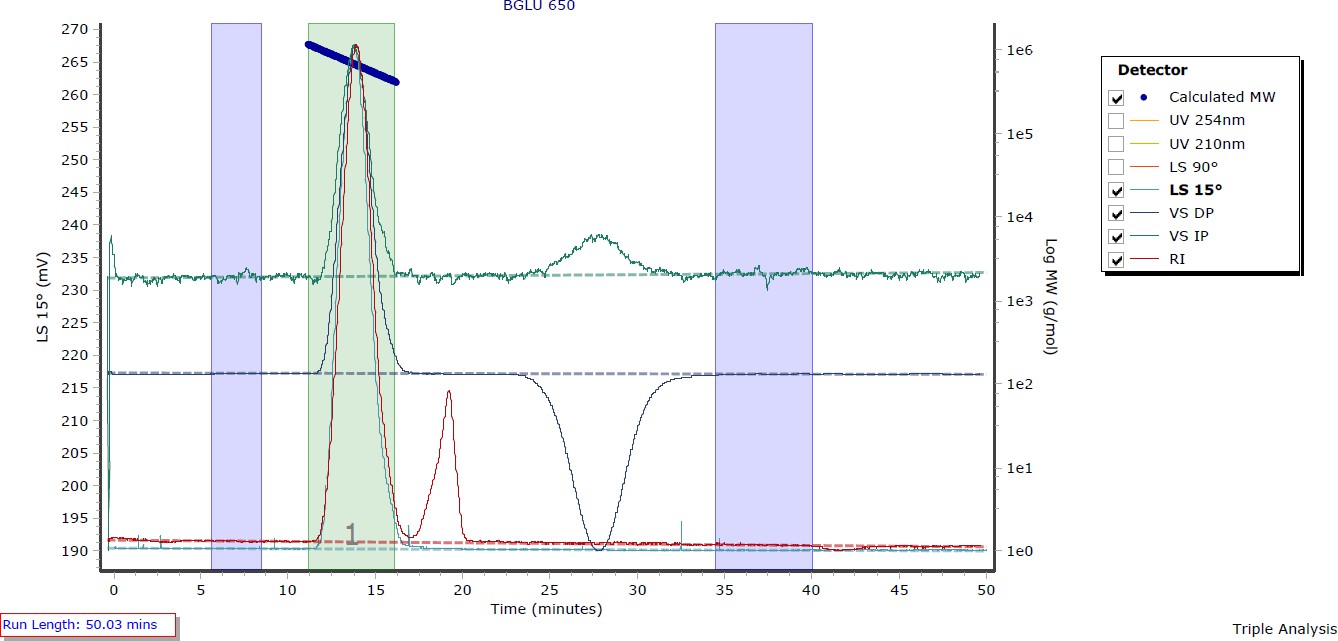


*MwControl Sample*

*β-glucanMwstandards(Megazyme,P-MWBGS)*
